# Improved protocols using sodium artesunate for the treatment of experimental cerebral malaria

**DOI:** 10.1128/aac.00154-26

**Published:** 2026-06-18

**Authors:** Melina Pedroso Merlone, Fabiana Gomes, Guilherme Salgado Sanches, Cláudio Tadeu Daniel-Ribeiro, Leonardo José de Moura Carvalho

**Affiliations:** 1 Laboratório de Pesquisa em Malária, Fundação Oswaldo Cruz (Fiocruz), Instituto Oswaldo Cruz, Rio de Janeiro, Brazil; The Children's Hospital of Philadelphia, Philadelphia, Pennsylvania, USA

**Keywords:** cerebral malaria, artesunate, therapeutics

## Abstract

Artemether and artesunate are effective antimalarial drugs in rescue therapies of *Plasmodium berghei* ANKA (PbA)-infected mice with experimental cerebral malaria (ECM), with a superiority for artemether among the single daily dosing schemes. The present study aimed to optimize the efficacy of artesunate treatment protocols using two daily dosing or slow continuous delivery schemes. PbA-infected mice were treated with four different artesunate protocols on day 5 post-infection (5 dpi). Protocol G4 (single intraperitoneal (IP) loading dose of 20 mg/kg followed by continuous delivery for 3 days with osmotic pumps) was superior to the other artesunate groups, with a 97.2% ± 0.86 reduction of parasitemia in 24 h, but recrudescence ensued in 50% of treated animals. Protocol G1 (loading dose of 40 mg/kg followed by 20 mg/kg twice a day for 5 days) led to a 90.5% ±5.39 decrease in parasitemia 24 h after the first dose without recrudescence. A variant of the G1 protocol, with two doses of 40 mg/kg in the first day, resulted in a survival rate of 42% in ECM-rescuing experiments, with ~30% decreases in hematocrit, hemoglobin, and red blood cell count 24 h after the first dose. Infected mice on 5 dpi showed severe thrombocytopenia (196 ± 118.3 platelets/μL; uninfected controls: 1,291 ± 256.4 platelets/μL), and artesunate treatment had little impact on these numbers 24 h after the first dose. In conclusion, two daily doses IP or via slow continuous delivery improve the efficacy of artesunate treatments compared to single-daily dose schemes and are suitable for ECM adjunctive therapy studies.

## INTRODUCTION

Cerebral malaria (CM) is a severe complication of *Plasmodium falciparum* malaria, which is responsible for the majority of malarial deaths (610,000 in 2024) (1). Intravenous artesunate has been determined as the most efficacious intervention in cases of severe malaria, including CM, being superior to intravenous quinine (2, 3). However, even with the best available conditions during clinical trials, the recorded mortality in these studies was 8% for all cases of severe malaria and 20% in cases of CM. In resource-limited settings, which is the common situation in most hyperendemic malaria areas in Africa where CM incidence is high, mortality can be even higher. Therefore, adjunctive interventions that help improve outcomes and decrease mortality in CM are urgently needed, but none has been proven efficacious thus far (4).

The murine model of cerebral malaria (CM), or experimental cerebral malaria (ECM), using *Plasmodium berghei* ANKA infection in the susceptible C57BL/6 mouse strain, is a valuable tool to explore the pathogenesis and therapeutics of human CM (5–8). Although some differences in pathology are well documented in relation to the human disease (9, 10), the large number of similarities makes it suitable for several types of investigation (11, 12). One of the potential uses for the model is to investigate adjunctive therapies that could then be tested in clinical trials, helping improve outcome during treatment of CM with antimalarial drugs.

For this, well-defined, reproducible treatment protocols must be put in place to generate solid data that can be further explored. We have been using this model for several years and described a protocol for testing adjunctive interventions in combination with antimalarial treatments (13). In that study, quinine and artemisinin were shown to be inappropriate choices as quinine did not cause a rapid reduction of parasitemia, which is a basic condition for an effective treatment during severe disease, and artemisinin was unable to clear parasitemia within 5 days of treatment. Injectable artesunate at single daily doses of 32 and 64 mg/kg of body weight induced a rapid decrease in parasitemia (65%–70% decrease) in the first 24 h; however, recrudescence was observed in both cases, especially with the 32 mg/kg dose scheme. The most effective drug was artemether, which given at 25 mg/kg, intraperitoneally (IP), once daily for 5 days caused the most striking decrease in parasitemia in the first 24 h (~95%), leading to parasite clearance in 48–72 h and no recrudescence. Although the antimalarial efficacy of artemether 25 mg/kg was superior to that of artesunate 32 mg/kg, both drugs showed good efficacy in rescuing mice with late-stage ECM (13). Since then, we have used this artemether protocol in our studies (5, 6, 14, 15).

Although artemether seems to be a better option for ECM-rescuing treatments, artesunate remains as an alternative for different reasons. First, since it is the drug of choice in human CM, investigators may want to stick to this drug trying to more closely mimic the clinical conditions. This argument is valid, although the difference in efficacy between artesunate and artemether, both in humans and in mice, is most likely not due to an intrinsic molecular feature but rather to the specific treatment schemes, formulation, and route of delivery (16–18). Artesunate is also preferred because it is water-soluble, making it easier to formulate, while artemether is lipid-soluble. Artemether use has also been associated with neurotoxicity in animal models, but no evidence of neurotoxicity has been observed during its use in severe malaria (18, 19). Another argument for artesunate is access. Because intravenous artesunate has been defined as the first-line drug for treatment of severe malaria worldwide, it is easier to have access to it than to injectable artemether. In Brazil, for instance, the Ministry of Health used to distribute both intravenous artesunate and intramuscular artemether to public hospitals, but now, only intravenous artesunate is distributed. Access to commercial sources for research purposes in Brazil requires import from international companies, which can take many months, and is subjected to hurdles such as delivery and customs restrictions. Therefore, developing alternative ECM-rescuing protocols using artesunate can facilitate the design and conduction of preclinical adjunctive therapy studies using the mouse model.

In the present study, we sought to describe a suitable protocol for rescuing mice with late-stage ECM using artesunate. Four different protocols to define antimalarial efficacy were used, and one of them is selected for checking ECM-rescuing efficacy and its effects on hematological parameters.

## MATERIALS AND METHODS

### Animals, parasite, and infection

C57BL/6 male and female mice between 10 and 16 weeks of age were provided by the Institute of Science and Technology in Biomodels (ICTB) of Fiocruz. The animals were placed with no more than five per cage, with free access to food and water, maintained in a room at a constant temperature of 21°C–22°C and a 12-h light/dark cycle. This study was approved by the Animal Use Ethics Committee from Fiocruz under license number L-007/2023.

Mice were infected intraperitoneally (IP) with *Plasmodium berghei* ANKA (PbA) expressing green fluorescence protein (GFP), which was kept in liquid nitrogen and thawed to room temperature before injection. After 4 to 5 days, blood was collected, and red blood cell count and parasitemia were assessed in order to infect the experimental groups with an inoculum of 1 × 10^6^ infected red blood cells. Parasitemia was followed since day 5 of infection by flow cytometry or by Giemsa/panoptic-stained thin smears of blood collected from the tail. Rectal temperature was also followed, measured with a rectal thermocouple probe suitable for small animals (Oakton Acorn Tm, Oakton Instruments, IL, USA).

### Study design, drugs, and treatment protocols

This study was conducted with two distinct approaches. First, to determine a protocol for optimal antimalarial efficacy, mice were infected with *P. berghei* ANKA, and treatment intervention was started on the 5th day post-infection (5 dpi). The reason is because on 5 dpi, the mice show a relatively high parasitemia (usually 6%–12%) but no neurological signs and, therefore, all treated mice survive, allowing to determine antimalarial efficacy with lower number of animals. Once the best protocol was determined, the second approach consisted of checking its efficacy in rescuing mice with late-stage ECM, with treatment starting on 6 dpi with animals showing full-blown ECM.

Artesunate was provided as larinate-60 (Ipca Laboratories Ltd., Mumbai, India; kindly donated by the Brazilian Ministry of Health) or artesunate (artesunic acid, kindly donated by Dr Nubia Boechat, Farmanguinhos, Fiocruz, Rio de Janeiro, Brazil). The powder was initially dissolved in 5% sodium bicarbonate and then diluted with saline 0.9% at final concentrations of 10 mg/mL, 5 mg/mL, and 2.5 mg/mL. Artemether was purchased from Sigma-Aldrich and dissolved in fractioned coconut oil (medium-chain triglyceride solution, MCT, Vitafor, São Paulo, Brazil). Mefloquine (Farmanguinhos, Fiocruz, Rio de Janeiro, Brasil) was dissolved with 10% dimethyl sulfoxide (DMSO) in saline 0.9% at a final concentration of 5 mg/mL.

### Treatment protocols for Approach 1: treatment starting on 5 dpi

Group 1 (G1): first dose of sodium artesunate 40 mg/kg, followed by other doses of 20 mg/kg, with two doses (100 μL IP) per day for 5 consecutive days;

Group 2 (G2): first dose of sodium artesunate 20 mg/kg and other doses of 10 mg/kg, with two doses (100 μL IP) per day for 5 consecutive days;

Group 3 (G3): first dose of sodium artesunate 20 mg/kg and other doses of 10 mg/kg, with two doses (100 μL IP) per day for 3 consecutive days, and, with the last dose of artesunate, a single dose of mefloquine 40 mg/kg was administered by gavage (200 μL);

Group 4 (G4): first dose of sodium artesunate 20 mg/kg (100 μL IP) and, right after, an Alzet osmotic pump (DURECT, Cupertino, CA, USA – model 1003D) was surgically implanted subcutaneously (scapulae), which delivered continuously 10 mg/kg/day of sodium artesunate for 3 days;

Group 5 (G5): artemether 20 mg/kg, single daily dose (50 μL IP) for 5 consecutive days.

In groups G1, G2, and G3, the interval between the first (morning) and second (afternoon) dose of the day was 8 h.

The rationale for choosing the doses described above was based on the following. A previous study showed that a single daily dose of artesunate at 64 mg/kg IP for 5 days was effective in rapidly decreasing parasitemia in *P. berghei* ANKA-infected mice, but recrudescence was observed. Half the dose (32 mg/kg) was even less effective in preventing recrudescence, and both doses were less effective than artemether 25 mg/kg (13). We assumed that two daily doses of artesunate would markedly enhance the efficacy, which would also allow to decrease the dose (then we defined 40 mg/kg and 20 mg/kg as first loading doses and 20 mg/kg and 10 mg/kg for the following doses over 5 days). For Group 3, we chose the lower dose of 10 mg/kg/day as we assumed that continuous delivery would be more effective. For the mefloquine dose, we used the same dose as used in Clemmer et al. (13), which provided a successful treatment in combination with artemether. In this study, artemether was used as a “positive” control, whose efficacy has been demonstrated in our previous studies.

### Implantation of the osmotic pump

The Alzet osmotic pump (DURECT, Cupertino, CA, USA – model 1003D) has a constant delivery rate of 1 µL/hour for 3 days and was filled with 100 µL of artesunate 10 mg/mL, allowing it to deliver 10 mg/kg/day of the drug. Right after receiving the first dose of artesunate IP, mice were anesthetized with isoflurane 5%. After losing reflections, a small incision was made on the back, slightly after the scapulae, and the pump was inserted with the delivery portal turned toward the head so as not to be affected by the healing wound. The incision was sutured, and an anti-inflammatory antibiotic cream (Neodexa F cream, Coveli) was applied on the wound. With the osmotic pump, the animals received a daily dose of 0.5 mg/kg subcutaneous buprenorphine. The pump starts to deliver the drug after 4 h under body temperature. After 72 h, the osmotic pump was removed, as recommended by the manufacturer.

### Treatment protocols for Approach 2: survival assay

Mice were infected, and starting on 5 dpi, the rectal temperature was measured. To be able to compare the efficacy of treatments in ECM, it is important that the stage of the disease is properly determined. A reliable and objective indicator of ECM progression is rectal temperature. Mice with ECM consistently present progressive hypothermia, and the more severe the hypothermia, the more severe the disease (20). For this reason, rectal temperature was established as a disease-stage indicator for ECM (14).

Three protocols were tested (after the results of Approach 1):

- On 6 dpi, mice with ECM received a first dose of sodium artesunate 40 mg/kg and other doses of 20 mg/kg, with two doses (100 μL IP) per day for a total of 5 consecutive days;
- On 6 dpi, mice with ECM received the first and second doses of sodium artesunate 40 mg/kg and other doses of 20 mg/kg, with two doses (100 μL IP) per day for a total of 5 consecutive days;
- Because mortality was high in one of the 6 dpi groups, a third protocol was evaluated that started treatment on the evening of 5 dpi (“5.5 dpi”); the first dose of 40 mg/kg IP was followed by doses of 20 mg/kg, with two doses (100 μL IP) per day for a total of 5 consecutive days.

These three groups were treated for 5 consecutive days (up to 10 dpi) and accompanied for more 6 days after ending treatment (up to 16 dpi).

### Hematological analysis

Mice were infected with *P. berghei* ANKA and randomly distributed into four groups for blood collection:

- Infected nontreated mice day 5: blood was collected on the morning of 5 dpi;
- Infected mice treated with one dose of 40 mg/Kg of sodium artesunate: mice were treated on the morning of 5 dpi and blood collected 6 h later;
- Infected mice treated with two doses of 40 mg/Kg of sodium artesunate: mice were treated on the morning and afternoon (8 h apart) of 5 dpi and blood collected 24 h after the first dose (morning of 6 dpi).
- Infected nontreated mice day 6: blood was collected on the morning of 6 dpi.

The blood collection procedure was terminal; therefore, a single sample was obtained from each animal. Uninfected animals also had blood collected to determine control parameters of hematocrit, hemoglobin, red blood cells (RBC), leukocytes, and platelet count. The animals were anesthetized with intramuscular ketamine and xylazine, blood was collected by cardiac puncture and placed in 500 µL EDTA tubes, and then they were subjected to euthanasia. The blood analysis was performed at ICTB-Fiocruz using a POCH-100iV Diff hematology analyzer (Sysmex).

### Statistical analyses

Data were analyzed using a statistical software package (GraphPad Prism 7.0, La Jolla, CA). The Shapiro-Wilk test was used to check the distribution among the tested groups. Data are reported as mean ± standard deviation, where values of *P*  < 0.05 were considered significant. Comparisons between two groups were performed using Student *t*-test and Mann-Whitney test, and multiple groups were compared using one-way ANOVA and Kruskal-Walis test. For survival analysis, the Mantel-Cox log-rank test was used.

## RESULTS

### Anti-parasite efficacy of different artesunate treatment protocols

Four different protocols of artesunate treatment were tested in mice infected with *P. berghei* ANKA. Treatment started on the 5th day post-infection (5 dpi), which is a time when parasitemia is relatively high, but mice did not yet develop the neurological signs, and treatment was continued for 3 (groups G3 and G4) or 5 (groups G1 and G2) days. Group G5, with the standard artemether treatment (20 mg/kg, single daily dose for 5 days), was included for comparison. Mice in the five groups showed levels of parasitemia (measured by flow cytometry) on 5 dpi (pre-treatment), ranging from a minimum of 4.9% to a maximum of 13.3% (mean ± standard deviation: G1: 8.1% ± 1.33; G2: 10.4% ± 1.67; G3: 9.18% ± 1.78; G4: 9.0% ± 2.32; G5: 10.7% ± 0.70). No differences in pretreatment parasitemia were observed between groups, except for group G2 showing a significantly higher parasitemia than group G1 (*P* = 0.0461).

**Fig 1:**
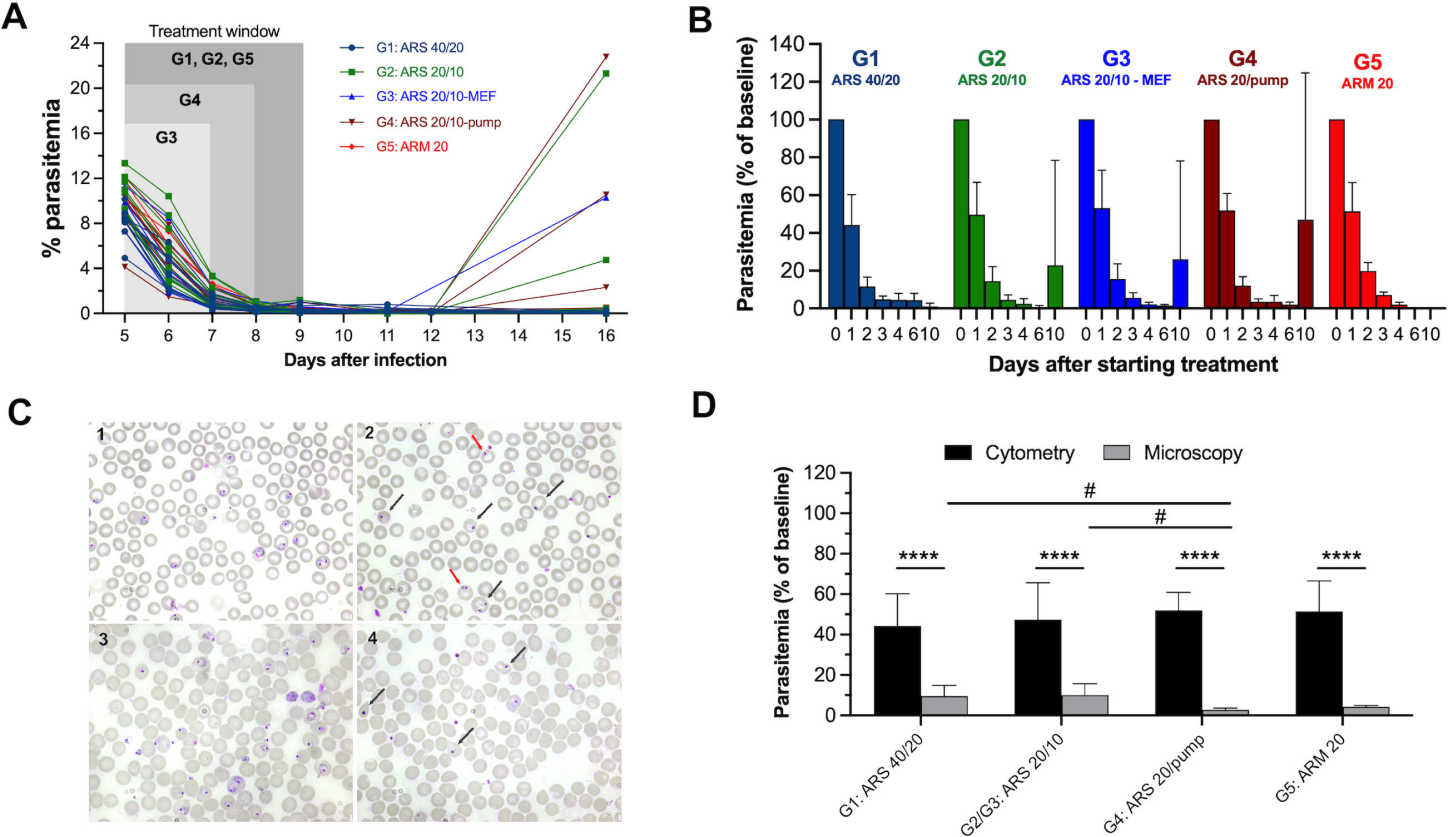
Efficacy of artesunate and artemether treatments on parasitemia in *Plasmodium berghei* ANKA-infected mice on 5 days post-infection (5 dpi). four artesunate protocols were tested, and a standard artemether protocol was used for comparison (see details at the end of the legend). (**A**) Parasitemia curves (GFP detection by flow cytometry) of individual mice treated with the different protocols; the shaded gray areas indicate the treatment windows (3–5 days). (**B**) Magnitude of parasitemia reduction measured by flow cytometry relative to baseline levels with different treatment protocols; there was no difference between groups. OBS: in Fig. 1B, the X axis refers to “days after starting treatment”; therefore, day 0 (zero) in Fig. 1B is equivalent to day 5 of infection (5 dpi) in Fig. 1A. The reason there is no variability in the day 0 bar in Fig. 1B is because the 5 dpi parasitemia for each animal was transformed as 100% baseline. And every value thereafter after starting treatment was calculated for each animal in relation to its own parasitemia. (**C**) Giemsa-stained thin blood smears from *Plasmodium berghei*-ANKA-infected mice on 5 dpi (pictures 1 and 3, pretreatment) and 24 h after first artesunate (picture 2) or artemether (picture 4) dose; parasites at pretreatment showed typical morphology, with well-defined blue-stained cytoplasm and red-stained nuclei; different stages, from ring to hemozoin-containing late trophozoites, are observed, consistent with *P. berghei* asynchronous development; thin smears prepared 24 h after the first dose show mostly dead or dying (condensed, pyknotic, or vacuolated) forms (black arrows), with only a few parasites with preserved morphology (red arrows)—only the latter were counted for parasitemia determination by microscopy. (**D**) Magnitude of parasitemia decrease (% of baseline) 24 h after the first dose of artesunate or artemether measured either by flow cytometry (GFP detection, black bars) or microscopy (Giemsa-stained thin smears, gray bars); groups G2 and G3 had the same treatment (artesunate 20 mg/kg first dose and 10 mg/kg second dose) in the first 24 h; therefore, mice from these groups were analyzed together; differences between the measurement methods (cytometry versus microscopy) were highly significant in all groups (*P* < 0.0001, ****); for microscopy measurements, group G4 showed superior performance (97.2% ± 0.86 reduction of parasitemia) compared to groups G1 (90.5% ±5.39, *P* = 0.0395, #) and G2/G3 (89.9% ± 5.60, *P* = 0.0164, #). Performance of the artemether group G5 (95.8% ± 0.73 reduction) was not significantly different from that of the artesunate groups. For comparing the different groups, Kruskal-Wallis followed by Dunn’s comparison test were performed. Student’s *t*-test was used for comparing cytometry versus microscopy data. Treatment details: Group 1 (G1; *n* = 10): first dose of sodium artesunate 40 mg/kg followed by other doses of 20 mg/kg, with two doses (100 μL IP) per day for 5 consecutive days; Group 2 (G2; *n* = 10): first dose of sodium artesunate 20 mg/kg and other doses of 10 mg/kg, with two doses (100 μL IP) per day for 5 consecutive days; Group 3 (G3; *n* = 5): first dose of sodium artesunate 20 mg/kg and other doses of 10 mg/kg, with two doses (100 μL IP) per day for 3 consecutive days and, with the last dose of artesunate, a single dose of mefloquine 40 mg/kg was administered by gavage (200 μL); Group 4 (G4; *n* = 8): first dose of sodium artesunate 20 mg/kg (100 μL IP) and, right after, an Alzet osmotic pump (DURECT, Cupertino, CA, USA – model 1003D) was surgically implanted subcutaneously (scapulae), which delivered continuously 10 mg/kg/day of sodium artesunate for 3 days; Group 5 (G5; *n* = 5): artemether 20 mg/kg, single daily dose (50 μL IP) for 5 consecutive days.

All treatments resulted in progressive reductions in parasitemia (by flow cytometry) over time (Fig. 1A). The first key information is the magnitude of decrease in parasitemia in the first 24 h because this is a critical factor in ECM-rescuing therapeutic studies. Since groups G1 and G3 share the same protocol (artesunate IP 20/10 mg/kg) on the first day of treatment, these two groups were analyzed together to determine the efficacy of this scheme in the first 24 h. The G1 protocol resulted in a 55.8% ± 15.97 reduction of parasitemia in the first 24 h, with similar outcomes in G2/G3 (52.6% ± 18.30), G4 (48.0% ± 8.86), and G5 (48.6% ± 15.11) (Fig. 1B). The differences between groups were not significant. However, parasitemia quantification by flow cytometry after treatment was shown to be inaccurate, at least in the first time point (24 h) after starting artesunate or artemether treatment. At this time point shortly after starting treatment, many dead or dying parasites possessing residual GFP are still circulating (Fig. 1C), but not yet eliminated by splenic clearance. Microscopic analysis of Giemsa-stained thin blood smears, which allows more accurate assessment of viable versus nonviable/dead parasites, revealed that the decrease in parasitemia at 24 h after starting treatment was more remarkable than that detected by flow cytometry. Indeed, the magnitude of the decrease in parasitemia after 24 h was 90% or above in all groups (Fig. 1D), compared to ~50% decrease observed by flow cytometry. By microscopy, the effectiveness of G4 treatment (single IP loading 20 mg/kg dose followed by slow continuous delivery for 3 days with osmotic pumps) was superior to that of the other artesunate groups, with a 97.2% ± 0.86 reduction of parasitemia in 24 h compared to 90.5% ±5.39 for G1 (*P* = 0.0395) and 89.9% ± 5.60 for G2/G3 (*P* = 0.0164). The performance of the artemether group G5 (95.8% ± 0.73 reduction) was not significantly different from that of the artesunate groups.

The follow-up by flow cytometry revealed a progressive reduction in parasitemia, reaching complete or nearly complete elimination of blood parasites 3–6 days after the start of treatment (Fig. 1B). On 16 dpi, meaning 7–9 days after the last dose (depending on the group), recrudescence partially occurred in some groups: 30% (3/10) of mice in group G2, 20% (1/5) mice in group G3, and 50% (4/8) of mice in group G4. No recrudescence was observed in groups G1 (0/10) and G5 (0/5). Therefore, a total of eight mice in all groups showed recrudescence by 16 dpi, with recrudescent parasitemias ranging from 0.4% to 22.8% (Fig. 1A).

The data show that the four different artesunate treatment protocols tested in this study were able to cause a rapid and efficient decrease in parasitemia, comparable to that of artemether. Protocol G1 was selected for the follow-up studies.

### Efficacy of artesunate IP twice a day to rescue mice with ECM

**Fig 2:**
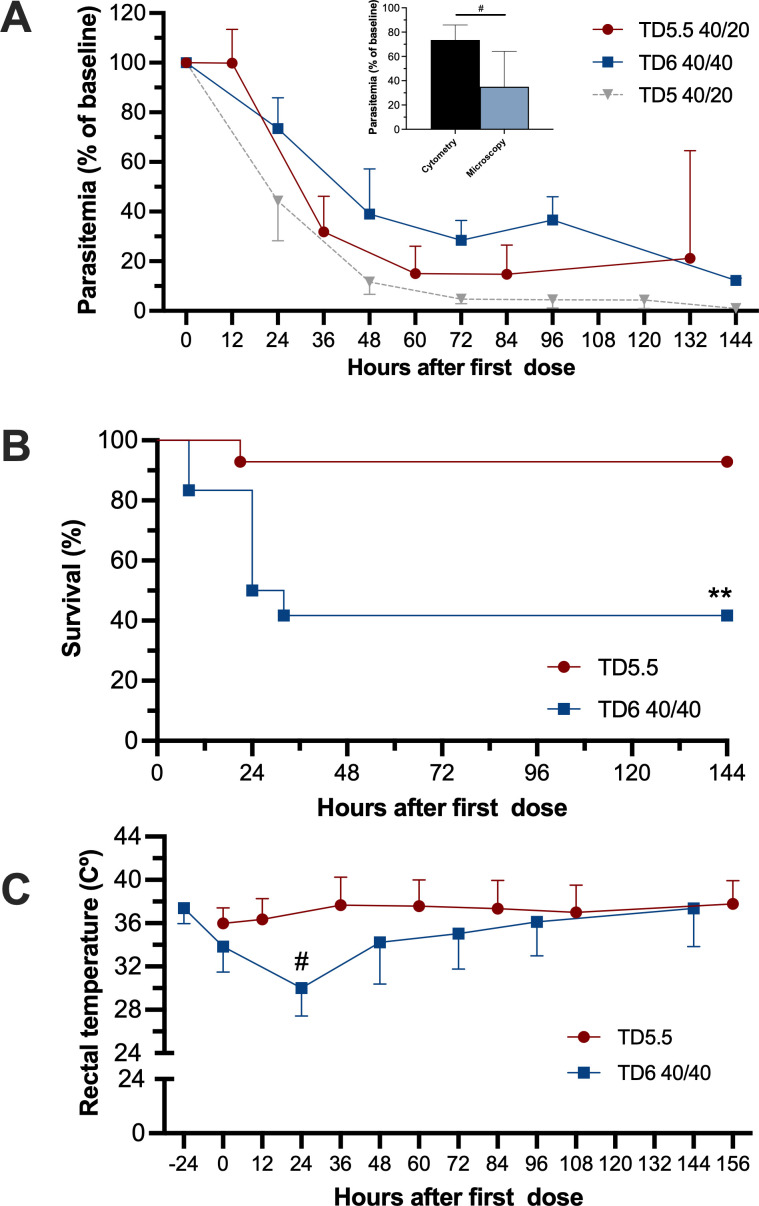
Efficacy of artesunate given intraperitoneally (IP) in rescuing mice with early- or late-stage experimental cerebral malaria (ECM). (**A**) Curves of parasitemia (flow cytometry) of artesunate-treated *Plasmodium berghei*-ANKA-infected mice before developing ECM (5 dpi, gray label; *n* = 10) or showing early-stage (5.5 dpi, red label; *n* = 14) or late-stage (6 dpi, blue label; *n* = 12) ECM. Protocol G1 (“40/20”: first dose of 40 mg/kg IP followed by doses of 20 mg/kg IP) was used to treat before ECM and early-stage ECM, whereas protocol G1 modified (“40/40”: first and second dose of 40 mg/kg IP followed by doses of 20 mg/kg IP) was used to treat late-stage ECM. The insert shows the parasitemia (% of baseline) 24 h after the first dose, with the protocol G1 modified measured by flow cytometry (26.5% ±12.39 reduction) versus microscopy (65.0% ±29.13 reduction; *P* = 0.0321, #). (**B**) Survival curves and (**C**) body temperature of mice with early-stage (5.5 dpi, red label; *n* = 14) or late-stage (6 dpi, blue label; *n* = 12) ECM treated with artesunate protocols G1 (“40/20”) or G1 modified (“40/40”); higher survival was observed with early-stage (5.5 dpi) treatment (*P* = 0.0070, **), and mice treated at late-stage (6 dpi) ECM showed lower body temperatures (*P* = 0.0409, #). Survival curves were analyzed using the log-rank test (Mantel-Cox) test, and rectal temperature data were analyzed with two-way ANOVA (mixed-effects model).

Once the efficacy of artesunate to decrease *P. berghei* ANKA parasitemia on 5 dpi was established under different protocols, the key question was whether the treatment is effective in rescuing mice with late-stage ECM (6 dpi). A prior study showed that artesunate given IP at a dose of 32 mg/kg once daily to mice with ECM resulted in a survival rate of 43% (13) and that artemether showed similar efficacy overall (46% survival), with a better performance within the first 24 h (64% versus 43%). A preliminary experiment was performed to establish the efficacy of artesunate in protocol G1 to decrease parasitemia in 24 h on 6 dpi. Surprisingly, the anti-parasite efficacy of protocol G1 on 6 dpi was much lower (only 11% decrease in parasitemia by flow cytometry) compared to the same protocol on 5 dpi (55.8% ± 15.97 reduction of parasitemia). Therefore, for the survival assessment, a modified G1 protocol was used, where the second dose on 6 dpi was adjusted to 40 mg/kg, followed by 20 mg/kg twice a day in the next 4 days. With this 40/40 mg/kg protocol on 6 dpi, the reduction of parasitemia in the first 24 h was 26.5% ±12.39 by flow cytometry (Fig. 2A) and 65.0% ±29.13 by microscopy (Fig. 2A— insert); still a poorer performance was observed compared to the G1 protocol applied on 5 dpi. Also, in this case, the full 5-day treatment protocol was unable to eliminate the parasites in the survivor animals. The modified 40/40 mg/kg protocol resulted in a 42% survival overall (50% survival in the first 24 h) of mice with late-stage ECM (body temperature: 33.8°C ± 2.35) (Fig. 2B).

An alternative protocol was also evaluated in which mice were treated not on the morning of 6 dpi (when they present with full-blown, late-stage ECM), but on the evening of 5 dpi (“5.5 dpi”), at an earlier stage of ECM development (body temperature: 36.0°C ± 1.43). Treating mice at this earlier stage of ECM resulted in a negligible effect on parasitemia by flow cytometry after 12 h and a 68.2% ± 14.29 decrease in 36 h (Fig. 2A) and a high survival rate (92%) (Fig. 2B). This high survival rate, however, makes this protocol unsuitable for the purposes of a model designed to assess the efficacy of adjunctive therapies in ECM because the antimalarial drug (artesunate) is highly efficacious on its own.

Treatment of mice with artesunate on 5.5 dpi prevented the aggravation of hypothermia and led to rapid recovery of body temperature, whereas mice with more severe hypothermia (full-blown ECM) treated on 6 dpi showed a deterioration of body temperature in the first 24 h after the first dose and a slow recovery to normal temperature in the survivors (Fig. 2C).

### Hematological parameters before and after treatment with artesunate

It has been previously shown that mice with ECM receiving artemether 20 mg/kg present a substantial (28%) decrease in hematocrit in the first 24 h in relation to pretreatment (6). Here, we investigated whether artesunate treatment induces similar hematological responses. Mice were infected with *P. berghei* ANKA, and whole blood was collected on 5 dpi (pretreatment), 6 h after one dose of artesunate 40 mg/kg and 24 h after two doses of artesunate 40 mg/kg (doses given at 0 h and 8 h). Blood was also collected from mice with ECM on 6 dpi (equivalent to 24 h after no treatment on 5 dpi).

**Fig 3:**
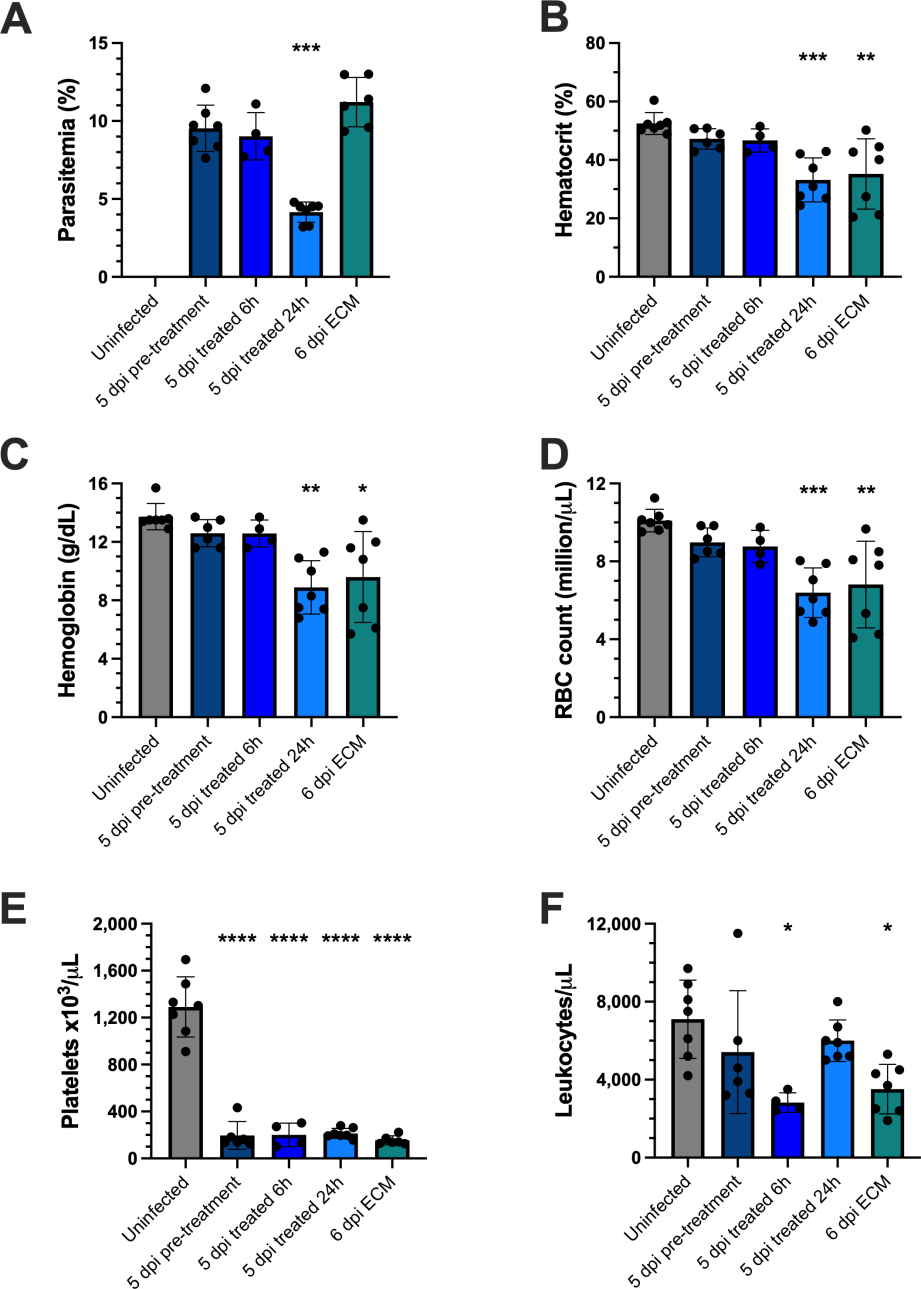
Hematological parameters in *Plasmodium berghei* ANKA-infected mice treated with artesunate protocol G1 modified (first and second dose of 40 mg/kg IP). Each parameter was measured in uninfected control mice (*n* = 7), in infected mice before (5 dpi; *n* = 6), 6 h (*n* = 4), and 24 h (*n* = 7) after treatment and in untreated mice with ECM on 6 dpi. (**A**) Parasitemia levels. (**B**) Hematocrit. (**C**) Hemoglobin levels. (**D**) Red blood cell count. (**E**) Platelet count. (**F**) White blood cell count. Artesunate-treated mice showed marked decreases in hematocrit (*P* = 0.0008, ***), hemoglobin levels (*P* = 0.0018, **), and red blood cell count (*P* = 0.0006, ***) 24 h after the first dose compared to uninfected controls. These decreases were similar to those observed in untreated mice on 6 dpi (although parasitemia levels in the latter were much higher). Infected mice showed severe thrombocytopenia on 5 dpi and 6 dpi (*P* < 0.0001, ****) compared to uninfected controls, and platelet counts did not recover 24 h after artesunate treatment. Infected mice showed leukopenia on 6 dpi (*P* = 0.0270, *), which was prevented by artesunate treatment. For comparing the different groups, Kruskal-Wallis followed by Dunn’s comparison tests were performed.

On 5 dpi (pretreatment), infected mice showed a mean 10% decrease in hematocrit (Htc: 47.2% ± 3.47) compared to uninfected controls (Htc: 52.5% ± 3.74) (Fig. 3B). Six hours after a single dose of artesunate 40 mg/kg IP, hematocrit was similar to pretreatment levels (46.7% ± 3.97). However, 24 h after receiving two doses of artesunate 40 mg/kg, hematocrit dropped further by 30% (Htc: 33.2% ± 7.54) compared to pretreatment. On 6 dpi, hematocrit levels in untreated infected mice were comparable to the levels observed in mice that received two doses of artesunate (Htc: 35.2% ± 12.03). Hemoglobin levels and RBC counts showed profiles similar to those of hematocrit (Fig. 3C and D).

*Plasmodium berghei*-infected mice showed marked thrombocytopenia already on 5 dpi, with an ~85% decrease in platelet counts per μL (196 ± 118.3) compared to uninfected controls (1,291 ± 256.4) (Fig. 3E). Treatment with two doses of artesunate on 5 dpi did not improve platelet counts 24 h later (213 ± 43.0). The treated group, however, showed a slightly better performance than untreated infected mice on 6 dpi (154 ± 37.8).

*Plasmodium berghei* ANKA-infected mice showed leukopenia, which was partially reversed by artesunate 24 h after treatment (Fig. 3F).

## DISCUSSION

In the present study, we sought to identify a suitable protocol for using sodium artesunate as a drug for rescuing mice with late-stage ECM. Data from previous studies comparing protocols with artesunate, artemisinin, artemether, artemether-mefloquine, and quinine at single daily doses revealed that artesunate could be used for fast parasite killing and ECM-rescuing but was inferior to artemether in that context (13). For this reason, we have established artemether as the drug of choice for evaluating adjunctive therapies using the ECM model (5, 6, 13–15). However, recent difficulties in having access to artemether prompted us to investigate optimized ECM-rescuing protocols using sodium artesunate. The data obtained here revealed that optimized dosing of artesunate resulted in improved performances compared to the protocols described in Clemmer et al. (13).

In treating human severe malaria, artesunate is considered superior to artemether (16–18). This superiority seems to be due to the fact that artesunate is administered intravenously, which guarantees that its effect is immediate. Because the half-life of artesunate is short, additional doses are given at 12 h and 24 h, ensuring sustained efficacy (21). On the other hand, artemether as an oil formulation given intramuscularly probably takes more time to be absorbed and is subjected to slower and more erratic release from the injection site, especially due to the depot effect. These same features of artesunate and artemether may help explain why, conversely, artemether seems to present better antimalarial efficacy over single daily dose artesunate in *P. berghei* ANKA-treated mice. In mice, usually the antimalarial treatment is given as bolus injection via the intraperitoneal route. Because artemether is formulated in coconut oil, when given intraperitoneally, it results in immediate action as initial absorption is fast, but very likely, it also has a depot effect, resulting in more prolonged action and hence better efficacy. On the other hand, given the short artesunate half-life, this strategy with a single daily IP dose is probably not the best approach to ensure sustained parasite killing.

Therefore, the rationale for improving the parasite-killing efficacy of artesunate in *P. berghei* ANKA-infected mice in this study relied on shortened dosing intervals or on continuous delivery to ensure proper exposure of the parasites to the drug. The G1 and G2 protocols had the same design, focused on the inclusion of a second daily intraperitoneal dose of artesunate, and differed in the doses: loading doses of 40 mg/kg versus 20 mg/kg and remainder doses of 20 mg/kg versus 10 mg/kg for a total of 5 days. Protocol G3 explored a shortened (3-day instead of 5-day) treatment strategy, but including a single oral mefloquine dose on day 3 prevents recrudescence. And protocol G4 was based on the idea of providing continuous artesunate delivery for 3 days using subcutaneously implanted osmotic pumps to overcome the pharmacokinetic shortcomings of bolus artesunate. All four protocols showed good efficacy in reducing parasitemia by microscopy in the first 24 h, with performances in line with that of the artemether single dose in this study and in Clemmer et al. (13). Among the four artesunate treatment protocols, protocol G4 (single IP loading 20 mg/kg dose followed by slow continuous delivery for 3 days with osmotic pumps) presented the best performance (~97% reduction of parasitemia in 24 h, comparable to single-dose artemether). However, this protocol also resulted in a higher incidence (50%) of recurrence after finishing treatment. The reason for this apparent discrepancy in immediate versus long-term efficacy is unknown, but one possibility is that continuous delivery of artesunate after a loading dose ensures continuous exposure of the parasites to the drug, making it more effective in the first 24 h. But sodium artesunate shows poor stability in aqueous solution (22, 23), and it is possible that in the osmotic pumps, it suffers considerable degradation over the 72 h period. Changes in the G4 protocol, for instance, with replacement of the pump every 24 h, or adding a single oral dose of mefloquine 3 days after the subcutaneous implantation of the pump, can be reasonable alternatives for combining best efficacy in the first 24 h (the critical period in ECM-rescuing therapies) with prevention of recrudescence.

The present study revealed that counting parasitemia based on the parasite’s GFP fluorescence by cytometry after treatment can be misleading. At 24 h after the first dose of artesunate or artemether, a decrease in parasitemia in the order of 48%–56% was observed by cytometry, whereas by microscopy, the decrease was in the order of 90%–97%. The reason for this discrepancy seems to be the residual fluorescence of circulating dead or dying parasites not yet eliminated by splenic clearance. Indeed, by microscopy, 24 h after the first dose, a few viable parasites (properly shaped and stained, with red nuclei and blue cytoplasm) were observed among a larger number of amorphous, pyknotic, “dot” remainders of parasites. These remainders of parasites probably still contain parts of cytoplasm with amounts of GFP and would, therefore, be counted in cytometry. It is, therefore, important that, in experiments like these, the parasitemia, especially in the first 24 h, is preferably counted by microscopy.

The single-daily dose protocols with artesunate in the study by Clemmer et al. (13) were unable to prevent recurrence of parasitemia shortly after, or even during, treatment. Recurrence was also observed in three (G2, G3, and G4) of the four protocols used in this study. In any case, contrary to the single-dose protocols of Clemmer et al. (13), in this study, recrudescence affected only part of the animals, with the highest incidence observed in the G2 and G4 groups (30% and 50% recurrence, respectively). The G2 group was designed as a group with lower doses of artesunate IP (20 mg/kg loading dose and 10 mg/kg remaining doses), which may bring benefits to avoid potential side effects, for instance, major decreases in hematocrit. This protocol showed efficacy in reducing parasitemia in the first 24 h comparable to the G1 group, which used doses twice as high. Although some recurrence occurred, depending on the study design, it can still be a good alternative. It is worth mentioning that the mice in group G2 (lower dose) showed higher parasitemia levels (10.4% ± 1.67) than the mice in group G1 (higher dose; 8.1% ± 1.33, *P* = 0.0416), and this may have affected the recurrence outcomes.

The G1 protocol (loading dose of artesunate 40 mg/kg followed by two daily doses of 20 mg/kg for 5 days) was picked for additional studies, in particular its ECM-rescuing efficacy, as it showed good efficacy in decreasing parasitemia in 24 h and no recurrence was observed. However, this protocol failed to induce a marked decrease in parasitemia when given to mice on 6 dpi in a preliminary experiment. Therefore, one change was made in the protocol: doubling the second dose on the first day of treatment to 40 mg/kg. This change improved performance, but it remained inferior to the efficacy observed on 5 dpi. The reason for this poorer performance of artesunate in mice with ECM compared to infected mice before ECM development is unknown. ECM animals were hypothermic (33.8°C ± 2.35 on 6 dpi), and body temperature takes time to recover in the survivor animals; hence, it is conceivable that parasites become less metabolically active, resulting in decreased susceptibility to the antimalarial drug, affecting efficacy. Alternatively, mice with ECM on 6 dpi may have impaired splenic parasite clearance capacity compared to pre-ECM on 5 dpi. Even with this impaired parasite-killing performance, this modified G1 protocol resulted in a 42% survival, which is similar to the performance observed with artemether (5, 6, 13–15), and it indicates that this protocol can be used in ECM-rescuing experiments.

We also tried the G1 protocol starting ECM treatment earlier on 5.5 dpi (evening of 5 dpi) instead of the morning of 6 dpi, but at this early stage of the neurological syndrome, the antimalarial treatment is very effective, resulting in >90% survival. This high survival precludes the use of this protocol in adjunctive therapy studies because artesunate *per se* is highly effective, making adjunctive therapy irrelevant or, at least, making it very hard to demonstrate its efficacy.

We moved then to establish the effects of artesunate treatment on hematological parameters, which affect pathogenesis and the recovery of animals rescued from ECM. The modified G1 protocol (two doses of artesunate at 40 mg/kg in the first day of treatment) was used in this case. The study by Gul et al. (6) showed that a single dose of artemether 20 mg/kg intraperitoneally given to mice with ECM on 6 dpi induced a substantial (~30%) decrease in hematocrit in 24 h. Here, we show that two doses of artesunate had similar effects, with hematocrit decreasing~30% 24 h after the first dose in mice treated on 5 dpi. Similar figures were observed for hemoglobin levels and red blood cell counts. At the first dose, parasitemia was 9.5% ± 1.48; therefore, it is unlikely that the marked decreases in hematocrit/hemoglobin levels/red blood cell counts were solely due to the elimination of parasitized red blood cells and probably involves mechanisms of uninfected red blood cell destruction that remain to be elucidated.

Also, mice with ECM on 6 dpi showed severe thrombocytopenia, with circulating platelet counts decreasing ~90% compared to control healthy mice, and artemether treatment did not change this number in 24 h (6). We show here that severe thrombocytopenia is observed already on 5 dpi in *P. berghei* ANKA-infected mice, with an ~85% decrease in platelet counts per μL compared to uninfected controls. Again, these numbers did not alter significantly 24 h after artesunate treatment, but it is noteworthy that platelet counts 24 h after artesunate treatment were slightly but significantly higher than platelet counts in mice left untreated (measured on 6 dpi). These data suggest that although antimalarial treatment *per se* does not rapidly impact on the reversal of thrombocytopenia, but it stops it from aggravating. Strong activation and sequestration of platelets seem to play important roles in the pathogenesis of human and experimental cerebral malaria (24, 25), and survivor ECM mice rescued with a combination of artemether and whole blood transfusion showed a partial recovery in circulating platelet counts 24 h after intervention (6). Circulating platelet counts and markers of platelet activation may, therefore, be relevant parameters to monitor in studies focused on describing potential adjunctive therapies in the ECM model. Similarly, artesunate treatment led to reversal of leukopenia in 24 h. This finding may indicate that an effective antimalarial treatment is capable of rapidly decreasing splenic congestion and leukocyte sequestration observed during *P. berghei* ANKA infection (26, 27).

In conclusion, treatment protocols with artesunate given IP twice a day, or with a loading IP dose followed by slow continuous delivery, can lead to a rapid decrease in parasitemia, comparable to single daily doses of artemether. The antimalarial activity is decreased in mice with ECM and hypothermia, which may compromise therapeutic efficacy. Two 40 mg/kg doses of artesunate on the first day of treatment resulted in 42% survival, indicating that it can be used in ECM-rescuing studies, particularly those aimed at identifying adjunctive therapies for cerebral malaria. This treatment led to decreased hematocrit and had a negligible effect on thrombocytopenia in 24 h.
